# Habitual Tea Consumption and Risk of Cataracts: A Longitudinal Study

**DOI:** 10.7150/ijms.75774

**Published:** 2022-09-11

**Authors:** Chia-Wen Chang, Jia-In Lee, Chun-Yen Huang, Chun-Chi Lu, Yao-Hua Liu, Shu-Pin Huang, Szu-Chia Chen, Jiun-Hung Geng

**Affiliations:** 1 Department of Emergency Medicine, Kaohsiung Municipal Siaogang Hospital, Kaohsiung Medical University Hospital, Kaohsiung Medical University, Kaohsiung, Taiwan; 2 Department of Psychiatry, Kaohsiung Medical University Hospital, Kaohsiung, Taiwan; 3 Department of Internal Medicine, Kaohsiung Medical University Hospital, Kaohsiung Medical University, Kaohsiung, Taiwan; 4 Department of Urology, Kaohsiung Medical University Hospital, Kaohsiung Medical University, Kaohsiung, Taiwan; 5 Graduate Institute of Clinical Medicine, College of Medicine, Kaohsiung Medical University, Kaohsiung, Taiwan; 6 Department of Internal Medicine, Kaohsiung Municipal Siaogang Hospital, Kaohsiung Medical University, Kaohsiung, Taiwan; 7 Department of Internal Medicine, Division of Nephrology, Kaohsiung Medical University Hospital, Kaohsiung Medical University, Kaohsiung, Taiwan; 8 Faculty of Medicine, College of Medicine, Kaohsiung Medical University, Kaohsiung, Taiwan; 9 Research Center for Environmental Medicine, Kaohsiung Medical University, Kaohsiung, Taiwan; 10 Department of Urology, Kaohsiung Municipal Siaogang Hospital, Kaohsiung, Taiwan

**Keywords:** Epidemiologic study, Longitudinal study, Cataract, Tea, Ocular disease, Risk factors.

## Abstract

We aimed to investigate the association between habitual tea consumption and the risk of developing cataracts in a large community-based cohort study. We prospectively collected volunteers from 29 recruitment centers that were ≧ 55 years old with no history of cataracts at the beginning of the study. There were 12,080 participants with available information in our study and were divided into two groups according to habitual tea consumption; non-tea-drinking and tea-drinking groups. The mean age was 59 years. Compared to the non-tea-drinking group, the tea-drinking group had a significantly lower incidence of developing cataracts (15.5% vs 12.1%) during follow-up of 46 months. In multivariate Cox proportional hazards regression analysis, the relative risk (RR) of incident cataracts was lower in the tea-drinking group than the non-tea-drinking group (RR = 0.848; 95% confidence interval [CI] = 0.751 to 0.957). Participants with ≧ 2 cups per day were associated with almost 16% reduction in the risk of developing cataracts compared with the non-tea-drinking group (RR = 0.844; 95% CI = 0.741 to 0.961). Our study suggests that habitual tea consumption can reduce the incidence of cataracts and raises the possibility that the tea content may slow the progression of cataracts.

## 1. Introduction

Cataracts, which are caused by the opacity of the lens, remain the leading cause of blindness and visual impairment in the elderly worldwide. Studies indicate that cases of blindness due to cataracts were over 12 million in 1990 and estimated to reach 13.5 million in 2020.2 1, 2. In Taiwan, the prevalence of cataracts was over 50% in a population-based cross-sectional study and nearly half (45%) of visual impairment was attributed to cataracts 3, 4. Age is a major risk of this multi-factorial disease which is also associated with sex, genetic predisposition, diabetes mellitus, smoking, alcohol, trauma, steroid use and exposure to UVB radiation 5, 6. Although cataract-related vision loss can be corrected by surgery, it is recognized as a huge burden and ophthalmological public health issue in an aging society due to costly medical expenses and postoperative complications. It has been calculated that delaying the onset of cataracts by 10 years would halve its incidence and therefore reduce the need for surgery 7. Therefore, it is crucial to identify the protective factors or seek the preventive intervention that can reduce the incidence or delay the onset of cataracts.

Tea, one of the most commonly consumed beverages in many countries, contains an abundance of bioactive compounds such as catechins, theanine-L and caffeine. These compounds exert antioxidant and anti-inflammatory properties which have been reported to have benefits in cardiovascular diseases 8, 9, metabolic syndrome, neurodegenerative disorders, ophthalmologic diseases and cancer prevention 10. However, studies regarding the impact of tea consumption on age-related cataracts remain limited. Our study aims to examine the temporal relationship between tea consumption habits and the risk of age-related cataracts through both the large-cohort study and long-term follow-up.

## 2. Methods

### 2.1. Study Population

**Figure 1:**
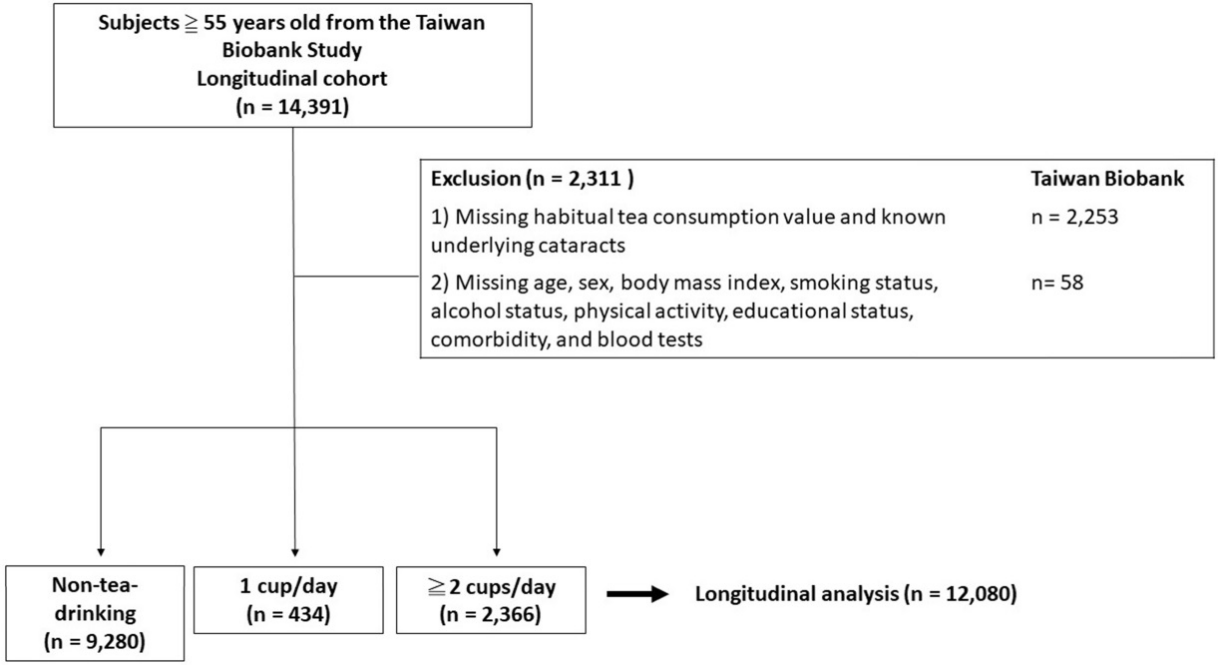
Study participants were classified by the status of their habitual tea consumption.

All participants were collected from 29 community-based recruitment sites in Taiwan as described in previous studies 11-13. Briefly, the cohort consisted of more than 20,000 volunteers aged 30 to 70 who underwent complete questionnaires, blood tests and long-term follow-up. In the present study, we collected participants ≧ 55 years old with no history of cataracts at the beginning of the study. A total of 12,080 subjects were recruited for the analysis (**Figure 1**). All subjects received regular follow-up every 2 to 4 years from the time point of participation to 2019. All the participants signed the informed consent and all researchers followed the Declaration of Helsinki. This study was approved by the Institutional Review Board of Kaohsiung Medical University Hospital (KMUHIRB-E(I)-20210058).

### 2.2. Habitual tea consumption Assessments

At first, participants were asked: “Do you consume tea regularly?” Subjects who consumed tea regularly were allocated as the tea-drinking group; others were assigned as the non-tea-drinking group. Subjects in the tea-drinking group were further asked “How many cups of tea do you consume every day?” Based on tea consumption, we divided them into “none”, “one cup per day”, and “≧ two cups per day” groups.

### 2.3. Assessment of cataracts

At the beginning and at each follow-up, subjects were asked: “Have you been diagnosed with cataracts?” The development of self-reported diagnosed cataracts was defined as subjects responding “Yes” to this question.

### 2.4. Statistical Analyses

In the present study, participants were divided into non-tea-drinking and tea-drinking exposure groups. Independent t tests and chi-square tests were used to compare the differences between the two groups. Univariate and multivariate Cox proportional hazards regression models were used to estimate the crude and adjusted relative risk (RR) and 95% confidence interval (CI) for the association between habitual tea consumption and the risk of developing cataracts. All the analyses were performed by using SPSS 20.0 (IBM Corp, Armonk, NY, USA) and R version 3.6.2 (R Foundation for Statistical Computing, Wien, Austria). A *P* value < 0.05 was considered statistically significant.

## 3. Results

### 3.1. Clinical characteristics of the study participants

Of the 12,080 recruited volunteers, mean ages were 59±4 years. Thirty-six percent of them (N = 4,353) were male and 23% (N = 2,800) were assigned to tea-drinking group (**Table 1**). Adults with habitual tea consumption tended to be male gander with higher rates of smoking, drinking, past history of diabetes mellitus, past history of gout, and a higher level of serum white blood cell count, red blood count, hemoglobin, triglyceride, uric acid, creatinine and body mass index, compared to those without habitual tea consumption (**Table 1**).

### 3.2. Habitual tea consumption was associated with a lower risk of development of cataracts

During a mean follow-up of 46 months, the participants with habitual tea consumption presented a significantly lower rate of incident cataract development (12.1%, N=339) compared to those with no habitual tea consumption (15.5%, N=1,441). In univariate Cox proportional hazards regression analysis, age, sex, smoking, drinking, physical activity, educational status, history of hypertension, history of diabetes mellitus, history of dyslipidemia, red blood count, serum hemoglobin, albumin, hemoglobin A1c, total cholesterol and uric acid were significantly associated with incident cataract development (**Table 2**). In the tea-drinking group, the relative risk of incident cataracts were lower than for the non-tea-drinking group (RR = 0.780; 95% CI = 0.693 to 0.878, *P* value = <0.001) (**Table 2**). After adjusting for confounders, male gender, higher educational status, higher levels of red blood count and habitual tea consumption were four independent protective factors for the development of cataracts (**Table 3**). Conversely, older age, physical activity, history of diabetes mellitus, and history of dyslipidemia were related to a higher incidence of cataracts (**Table 3**).

### 3.3. Association between daily cups of tea consumption and the development of cataracts

To further evaluate the dose-response effects of habitual tea consumption on the development of cataracts, we divided participants based on daily cups of tea consumption into three groups: non-tea-drinking, one cup per day, and ≧ two cups per day (**Table 4**). In multivariate Cox proportional hazards regression analysis, those that consumed one cup of tea per day (RR = 0.870; 95% CI = 0.666 to 1.136, *P* value = 0.307) or ≧ two cups of tea per day (RR = 0.844; 95% CI = 0.741 to 0.961, *P* value = 0.010) were associated with a lower risk for incident cataracts compared with those who did not consume tea regularly (**Table 4**).

### 3.4. Association between the types of tea and the development of cataracts

We also examined the associations between types of tea and the development of cataracts. We divided participants based on types of tea into two groups: fully-fermentation, and non-fermentation/semi-fermentation (**Supplementary Table 1**). In multivariate Cox proportional hazards regression analysis, those that consumed non-fermentation/semi-fermentation tea (RR = 0.836; 95% CI = 0.735 to 0.951, *P* value = 0.006) were associated with a lower risk for incident cataracts compared with those who did not consume tea regularly, but not for those who consumed fully-fermentation tea (OR = 0.955; 95% CI = 0.707 to 1.290, *P* value = 0.764) (**Supplementary Table 1**).

## Discussion

The present study is the first large community-based, longitudinal study to examine the association between habitual tea consumption and incident cataracts. Our results demonstrated a statistically significant decrease in the incidence of cataracts associated with tea consumption. A dose-response effect was also noted, which showed that ≧ two cups per day of tea consumption lowered almost 16% risk of developing cataracts compared with the non-tea-drinking group in later life.

Tea has been shown to have beneficial effects against a variety of ocular diseases including glaucoma, age-related macular degeneration, uveitis and ocular surface inflammation through cellular, animal, and human experiments 14. Kumar et al. demonstrated an anti-cataract effect of epigallocatechin-3-gallate (EGCG), a major active constituent of green tea through inhibition of crystallin aggregation *in vitro* 15. Thiagarajan et al. and Gupta et al. also have presented *in vivo* studies which suggested that green and black tea extracts led to a retardation of the progression of lens opacity via counteracting oxidative stress in selenite-induced cataracts in rats 16. However, epidemiologic evidence on the relationship between tea consumption and risk of cataract is scanty and controversial. While Robertson et al. noted that consumption of five or more cups of tea per day was inversely associated with cataracts 17, Tavani et al. hampered the perspective in light of the non-significant result from a case-control study 18. One cross-sectional study recently indicated that a tea-drinking habit was associated with a reduced risk of age-related cataracts. Due to the unavailability of large-cohort and long-term studies, we conducted this longitudinal study to verify the association between tea consumption and the development of cataracts.

Though there is mounting evidence from *in vitro* and *in vivo* studies that tea polyphenols could be beneficial in disease prevention or treatment, the exact dose-response relationship needs further confirmation. Observational research has found that tea consumption of two to three cups daily is associated with a reduced risk of heart disease, stroke, and type 2 diabetes 19. Additionally, three to five cups of green tea per day accounts for a minimum of 250 mg of catechins and is viewed as proper drinking in chemoprevention 20, 21. To investigate the dose-response effect of tea in reducing the cataract risk, the aforementioned analysis indicated that people drinking five more cups of tea per day for a period of five years had a significant reduction in cataract risk by 61% 17. Another study showed that average daily tea intake of more than two cups (one cup equal to 250 mL) at a moderate concentration (about 3-4 grams in 250 mL water) may inhibit age-related cataracts 22. In accordance with previous literature, our findings also support a dose-dependent effect of tea in cataract prevention and suggest that drinking two or more cups (one cup equal to 237mL) of tea per day notably decreased the incidence of age-related cataracts (RR=0.844; 95% CI = 0.741 to 0.961).

There are three major types of tea differing in manufacturing process and fermentation degrees, generally divided into green tea (non-fermented), oolong tea (semi-fermented), and black tea (fully-fermented). Catechins like (-)-epigallocatechin-3-gallate (EGCG), (-)-epigallocatechin (EGC), (-)-epicatechin-3-gallate (ECG) and (-)-epicatechin (EC) are the major polyphenols in green tea, with EGCG accounting for 50-70%. They are oxidized and dimerized during fermentation in both theaflavins (TF) and thearubigins, which are the two major polyphenols of black tea. Oolong tea, whose consumption was mostly confined to China and Taiwan, contains a mixture of catechins, TF and thearubigins 23. To compare the impact of different types of tea, it was demonstrated that TF and thearubigins, which were more abundant in black or oolong tea, possess relative antioxidant activity compared to catechins 24. Previous epidemiological studies also indicated that green tea and black tea are both associated with a significant reduction in age-related cataracts 16, 22, 25. In our study, the non-fermented and semi-fermented tea consumption group had a negative association with incidence of cataracts but a null association with the fully-fermented tea consumption group. Collaborating evidence from experimental and clinical studies inferred that the anti-oxidant effects of tea constituents may not be altered during fermentation, but the proportion of active compounds may decrease. We have made a generalization that different types of tea exert similar anti-cataract effects but the effective dose of individual tea needs further validation.

Multiple risk factors contribute to the development of cataracts, with age being the most important. In addition to old age, other individual factors such as females and low socioeconomic groups are susceptible to cataracts. Various epidemiologic studies suggest that lifestyle factors such as alcohol consumption, ultraviolet exposure, cigarette smoking and high carbohydrate diets are related to cataractogenesis. In addition, cataracts have been associated with many systemic diseases mainly diabetes mellitus, hypertension, obesity, chronic kidney disease and autoimmune disease 26-32. Consistent with previous research, our study adds to a growing body of recent epidemiological reports supporting that the an older age, female group, physical activity, history of diabetes mellitus, and history of dyslipidemia were related to a higher incidence of cataracts.

Tea has been considered as a potential option to delay or prevent cataract progression via antioxidant effects. To our knowledge, all three main types of age-related cataracts including nuclear, cortical, and posterior subcapsular cataracts were clearly associated with a direct insult of oxidative stress due to reduced levels of antioxidants, antioxidant enzymes, and proteases in aged lenses 33. The main bioactive constituents of tea extract are catechins which are polyphenolic compounds that may quench reactive oxygen radicals such superoxide and hydrogen peroxide, hence inhibiting oxidative stress 25, 34, 35. In addition, cataract formation is associated with protein precipitation and crystallin aggregation which significantly increase in quantity with aging. EGCG, a major component of green tea catechin, efficiently blocks crystallin aggregation and prevents initiation of cataract formation 15, 16, 36.

This is the first longitudinal study with a relatively large sample size to analyze the temporal and causal effects of tea consumption habits and risk of cataract disease while adjusting for various possible confounding factors. However, our study has some limitations. Firstly, information regarding cataracts was collected using questionnaires instead of medical records or image evidence, thus participants were unable to precisely classify the different types of cataracts. However, this self-reported measure may be clinically related to significant visual impairment that accentuates the group's severity and impact on their quality of life. It is also hard to collect complete image evidence in large biomedical databases like the multiple experiments conducted at the foundation of the powerful UK biobank. Furthermore, it is reasonable to speculate that misclassification of cataracts may not notably interfere with the results since antioxidant properties of tea play a protective role in cataractogenesis of all three types of cataracts 33. Secondly, this study lacks detailed information about individual tea intake and preparation, thus the exact dose effects of different types of tea require further investigation. Nevertheless, it has been demonstrated in previous studies that all tea catechins in fermented and non-fermented tea are effective in cataract prevention 25, 34, 35. Thirdly, though we have extensively collected multidimensional health variables, it is not possible to completely exclude potential confounders. Some unmeasured covariates may lead to study bias but are complex in standardization and quantification due to incongruent exposure such as UV light exposure, dietary habits, micronutrition supplements and systemic medication use. Fourthly, participants enrolled were ethnic Taiwanese individuals who consumed less fully-fermented tea which might bias our results toward a null association due to insufficient sample size. Additionally, further studies are needed in order to determine whether the development of cataracts is related to genetic predisposition, and should validate our results in different races. Lastly, it calls for an extended observation period to draw a more prudent and robust conclusion.

## Conclusions

Our study suggests that habitual tea consumption is a protective factor for developing cataracts based on a longitudinal study. It highlights the possibility that the tea content may slow the progression of cataracts and further research is warranted.

## Supplementary Material

Supplementary table.

## Figures and Tables

**Table 1:** Clinical characteristics of the study participants classified by tea consumption (n = 12,080).

Characteristics	Total (n=12,080)	Non-tea-drinking group (n=9,280)	Tea-drinking group (n=2,800)	*p* value
Age, yr	59±4	59±4	59±4	0.633
Male	4,353 (36)	2,911 (31)	1,442 (52)	<0.001
Body mass index, kg/m^2^	24±3	24±3.5	25±3	<0.001
Smoking status, ever	2,855 (24)	1,821 (20)	1,034 (37)	<0.001
Alcohol status, ever	1,084 (9)	644 (7)	440 (16)	<0.001
Physical activity, yes	7,176 (59)	5,492 (59)	1,684 (60)	0.368
Education status, n (%)				0.013
≦Elementary	1,482 (12)	1,173 (13)	309 (11)	
Middle to High school	6,157 (51)	4,750 (51)	1,407 (50)	
≧Collage	4,441 (37)	3,357 (36)	1,084 (39)	
Hypertension	2,284 (19)	1,719 (19)	565 (20)	0.050
Diabetes	838 (7)	600 (7)	238 (9)	<0.001
Dyslipidemia	1,210 (10)	931 (10)	279 (10)	0.941
Gout	525 (4)	372 (4)	153 (6)	0.001
White blood counts, 10^9^/L	5.8±1.5	5.8±1.5	6.0±1.6	<0.001
Red blood counts, 10^12^/L	4.7±0.5	4.7±0.5	4.8±0.5	<0.001
Platelet, 10^9^/L	230±54	231±55	228±53	0.023
Hemoglobin, g/dl	13.9±1.3	13.8±1.3	14.1±1.4	<0.001
Albumin, g/dl	4.5±0.2	4.5±0.2	4.5±0.2	0.015
Hemoglobin A1c, %	5.9±0.8	5.9±0.8	6.0±0.9	<0.001
Total cholesterol, mg/dl	202±36	203±36	199±36	<0.001
Triglyceride, mg/dl	119±80	118±76	125±89	<0.001
Uric acid, mg/dL	5.6±1.4	5.5±1.4	5.9±1.4	<0.001
Creatinine, mg/dL	0.7±0.3	0.7±0.3	0.8±0.2	<0.001

**Table 2:** Parameters associated with incident cataract.

Parameters	Relative risk (95% CI)	*p*
Age (per 1 year)	1.096 (1.086 to 1.107)	<0.001
Male (*vs.* female)	0.783 (0.708 to 0.866)	< 0.001
Body mass index (per 1 kg/m^2^)	0.989 (0.975 to 1.003)	0.129
Smoking status, ever (*vs.* never)	0.746 (0.663 to 0.841)	<0.001
Alcohol status, ever (*vs.* never)	0.733 (0.609 to 0.883)	0.001
Physical activity, yes (*vs.* no)	1.272 (1.154 to1.402)	<0.001
Education status, Middle to High school (*vs.* ≦Elementary)	0.640 (0.561 to 0.729)	< 0.001
Education status, ≧Collage (*vs.* ≦Elementary)	0.695 (0.607 to 0.796)	< 0.001
Hypertension, yes (*vs.* no)	1.180 (1.054 to 1.321)	0.004
Diabetes mellitus, yes (*vs.* no)	1.528 (1.311 to 1.781)	<0.001
Dyslipidemia, yes (*vs.* no)	1.346 (1.173 to 1.546)	<0.001
Gout, yes (*vs.* no)	0.982 (0.780 to 1.235)	0.874
White blood counts (per 10^9^/L)	0.984 (0.954 to 1.015)	0.315
Red blood counts (per 10^12^/L)	0.728 (0.658 to 0.806)	<0.001
Platelet (per 10^9^/L)	0.999 (0.998 to 1.000)	0.098
Hemoglobin (per 1 g/dl)	0.914 (0.883 to 0.946)	< 0.001
Albumin (per 1 g/dl)	0.751 (0.606 to 0.930)	0.009
Hemoglobin A1c (per 1 %)	1.089 (1.036 to 1.146)	0.001
Total cholesterol (per 1 mg/dl)	0.998 (0.997 to 1.000)	0.007
Triglyceride (per 1 mg/dl)	1.000 (0.999 to 1.000)	0.598
Uric acid (per 1 mg/dl)	0.940 (0.908 to 0.974)	0.001
Creatinine (per 1 mg/dL)	0.933 (0.765 to 1.138)	0.496
Tea consumption, yes (*vs.* no)	0.780 (0.693 to 0.878)	<0.001

CI = Confidence interval.

**Table 3:** Parameters associated with incident cataract after adjusting for confounders.

Parameters	Relative risk (95% CI)	*p*
Age (per 1 year)	1.093 (1.082 to 1.105)	<0.001
Male (*vs.* female)	0.803 (0.685 to 0.941)	0.007
Body mass index (per 1 kg/m^2^)	-	-
Smoking status, ever (*vs.* never)	0.948 (0.814 to 1.105)	0.494
Alcohol status, ever (*vs.* never)	0.933 (0.764 to 1.141)	0.502
Physical activity, yes (*vs.* no)	1.114 (1.009 to1.231)	0.033
Education status, Middle to High school (*vs.* ≦Elementary)	0.815 (0.714 to 0.931)	0.003
Education status, ≧Collage (*vs.* ≦Elementary)	0.914 (0.793 to 1.052)	0.210
Hypertension, yes (*vs.* no)	0.975 (0.864 to 1.100)	0.681
Diabetes mellitus, yes (*vs.* no)	1.243 (1.026 to 1.507)	0.027
Dyslipidemia, yes (*vs.* no)	1.221 (1.058 to 1.411)	0.006
Gout, yes (*vs.* no)	-	-
White blood counts (per 10^9^/L)	-	-
Red blood counts (per 10^12^/L)	0.869 (0.771 to 0.979)	0.021
Platelet (per 10^9^/L)	-	-
Hemoglobin (per 1 g/dl)	0.986 (0.940 to 1.034)	0.560
Albumin (per 1 g/dl)	1.100 (0.877 to 1.379)	0.410
Hemoglobin A1c (per 1 %)	1.036 (0.970 to 1.106)	0.296
Total cholesterol (per 1 mg/dl)	0.999 (0.997 to 1.000)	0.059
Triglyceride (per 1 mg/dl)	-	-
Uric acid (per 1 mg/dl)	0.966 (0.929 to 1.004)	0.077
Creatinine (per 1 mg/dL)	-	-
Tea consumption, yes (*vs.* no)	0.848 (0.751 to 0.957)	0.007

CI = Confidence interval. Multivariate Cox regression model: adjustment for age, sex, smoking status, alcohol status, Physical activity, educational status, history of hypertension, history of diabetes mellitus, history of dyslipidemia, red blood counts, hemoglobin, albumin, Hemoglobin A1c, total cholesterol and serum uric acid.

**Table 4:** Relative risk for incident cataracts according to daily cups of tea consumed by participants.

Daily cups of tea consumed	No. of incident cataract cases / No. of Subjects (%)	Adjusted relative risk (95% CI)	*P* value
None	1,441/9,280 (15.5)	1.000 (Reference)	-
1 cup per day	56/434 (12.9)	0.870 (0.666 to 1.136)	0.307
≧ 2 cups per day	283/2,366 (12.0)	0.844 (0.741 to 0.961)	0.010
Per 1 cup / day	-	0.917 (0.859 to 0.978)	0.008

* One cup = 0.237 liter. CI = Confidence interval. Multivariate Cox regression model: adjustment for age, sex, smoking status, alcohol status, Physical activity, educational status, history of hypertension, history of diabetes mellitus, history of dyslipidemia, red blood counts, hemoglobin, albumin, Hemoglobin A1c, total cholesterol, and serum uric acid.
