# Supplementary material for: Habitual Tea Consumption and Risk of Cataracts: A Longitudinal Study
Source: Int J Med Sci. 2022 Sep 11;19(10):1596–602. doi: 10.7150/ijms.75774 (PMC9515688; doi:10.7150/ijms.75774)
Supplement: Supplementary file 1 — Supplementary table. [file ijmsv19p1596s1.pdf]

Supplementary table 1. Relative risk for incident cataracts according to types of tea (n=12,040*)

**Daily cups of tea consumed**	No. of incident cataract cases / No. of Subjects (%)	Adjusted relative risk (95% CI)	*P* value
None	1,441/9,280 (15.5)	1.000 (Reference)	-
Fully-fermentation	44/323 (13.6)	0.955 (0.707 to 1.290)	0.764
Non-fermentation and semi-fermentation	290/2,437 (11.9)	0.836 (0.735 to 0.951)	0.006

* Participants with adequate information of types of tea (We excluded 40 participants who did not have information about the type of tea they were drinking)

CI = Confidence interval.

Multivariate Cox regression model: adjustment for age, sex, smoking status, alcohol status, physical activity, educational status, history of hypertension, history of diabetes mellitus, history of dyslipidemia, red blood counts, hemoglobin, albumin, Hemoglobin A1c, total cholesterol and serum uric acid.
